# Unforeseen Heat: An Accidental Superficial Partial-Thickness Hand Burn Utilizing a New Convection Oven

**DOI:** 10.7759/cureus.42641

**Published:** 2023-07-29

**Authors:** Mehmed F Canatan, Ahmed N Canatan, Mustafa O Canatan

**Affiliations:** 1 Faculty of Medicine, Gaziantep University, Gaziantep, TUR; 2 Faculty of Medicine, Marmara University, Istanbul, TUR; 3 Faculty of Medicine, Near East University, Nicosia, CYP

**Keywords:** thermal injury, burn prevention, kitchen accidents, heat exposure, convection oven, accidental burn, superficial partial-thickness burn, hand burn, burn injury

## Abstract

Thermal burns remain a significant public health concern, and it is crucial to understand the potential risks associated with everyday activities involving heated objects or surfaces. It has been found that males have a higher susceptibility to hand burns, but when it comes to burns caused by hot liquids, females tend to be more frequently affected. Cooking remains the predominant activity associated with a higher incidence of accidental hand burns. Prompt medical attention and appropriate management are essential in mitigating the severity of burn injuries. This case report presents a previously healthy 55-year-old female that sustained a superficial partial-thickness burn to the dorsal aspect of her right hand while preparing a meal in her kitchen with her brand-new convection oven. This was her first time using a convection oven, unaware of the fans and rapidly circulating hot air within the oven. We discuss the need for a comprehensive approach to wound care, including topical antimicrobial agents, dressings, pain management, and monitoring for potential complications to achieve favorable outcomes and minimize long-term sequelae. Prevention remains the cornerstone in reducing burn injuries. Awareness campaigns, safety guidelines, and educational initiatives aimed at promoting responsible handling of hot objects should be implemented. Simple preventive measures, such as using appropriate protective equipment such as oven mitts, being mindful of oven temperatures, and maintaining a safe distance from heated surfaces, can significantly reduce the risk of household thermal burns.

## Introduction

Burn injuries are common and can arise from various sources, including friction, extreme temperatures, radiation, chemicals, or electricity [1]. However, the most common cause of burn injuries is thermal burns as a result of exposure to heat from hot liquids, solids, steam, or fire [1]. A considerable proportion of individuals with burn injuries face the specific involvement of their hands, either as an isolated injury or as part of a broader burn condition [2]. Generally, males have a higher susceptibility to hand burns, but when it comes to burns caused by hot liquids, females tend to be more frequently affected [3]. Cooking stands out as the predominant activity associated with a higher incidence of accidental hand burns [3]. While some hand burns may present as mild, others can be severe and require specialized treatment at a burn center [2]. Unfortunately, a significant subset of patients experience complications, such as contractures, hypertrophic scars, and neuropathy, greatly affecting their overall well-being [4]. A fundamental principle in modern burn care is that the majority of burn injuries can be avoided through preventive measures [5]. It is of utmost importance to establish thorough nationwide programs for burn prevention education, which should cover critical aspects, such as promoting the use of anti-burn equipment like oven mitts in the kitchen, raising awareness about water temperature control in households, and encouraging the replacement of outdated heating equipment [3]. Burns are classified by considering both the depth of the injury and the percentage of total body surface area (TBSA) involved [6]. Based on the depth, burns can be categorized into five classifications: superficial, superficial partial thickness, deep partial thickness, full thickness, and deep full thickness, with each type of burn presenting its own set of unique characteristics and ultimately requiring different modalities of wound care and treatment [7]. For the purposes of this case report, we will be focusing our attention on superficial partial-thickness burns on the hand. The treatment of superficial partial-thickness thermal burns of the hand involves gentle wound care, including cleaning the burned hand with clean water, avoiding scrubbing, and leaving blisters intact [2]. Topical antimicrobial agents, such as silver sulfadiazine, bacitracin, mupirocin, or fusidic acid, may be applied to prevent infection and promote moist wound healing [8,9]. Non-adherent dressings are used to protect the wound, minimize pain during dressing changes, and promote healing [10]. Herein, we present a case report of a superficial partial-thickness burn of the dorsal aspect of the right hand that developed following sudden and unexpected contact with the intense heat emitted by a convection oven. We emphasize the importance of safety guidelines, awareness, and practicing simple yet effective preventive measures, such as wearing appropriate protective equipment.

## Case presentation

**Figure 1:**
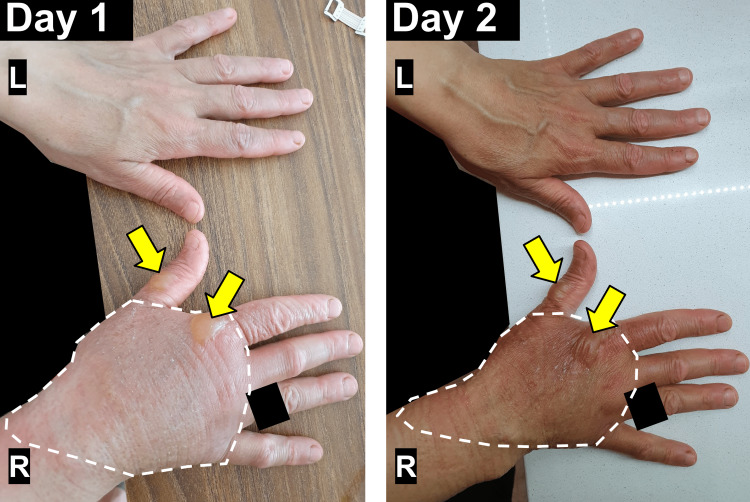
Superficial Partial-Thickness Hand Burn (Days One and Two Post-Incident). The superficial partial-thickness hand burn injury on days one and two post-incident can be seen on the dorsal aspect of the right hand (R=right hand, L=left hand). The affected area exhibits mild darkening and discoloration, accompanied by moderate edema evidenced by the disappearance of superficial veins (white dashed line). Blister formation is observed at the base of the first and second fingers of the right hand (yellow arrows). The surrounding skin appears intact without signs of deeper tissue involvement. Identifying features of the patient including clothing and rings have been blacked out upon their request.

In the presented case, a previously healthy 55-year-old female sustained a superficial partial-thickness burn to the dorsal aspect of her right hand while preparing a meal in her kitchen with her brand-new convection oven. The patient was previously using a small conventional oven that did not have fans to circulate the heat. This was her first time using a convection oven and was therefore unaware of the fans and rapidly circulating hot air within the oven. As she swiftly reached into the preheated oven to retrieve a baking dish, the dorsal aspect of her right hand got exposed to extremely hot air circulating in the convection oven, leading to immediate pain but no visible injury. The patient applied ice packs and did not feel the need to seek medical attention immediately post-incident due to no apparent physical changes in the external skin; this highlights the importance of timely evaluation and treatment for thermal burns. The patient went on with her day as if nothing had happened. However, the patient recounts waking up at three in the morning that very night with intense pain and edematous swelling of the dorsal aspect of the right hand, accompanied by blister formations at the base of the first and second fingers (Figure 1).

**Figure 2:**
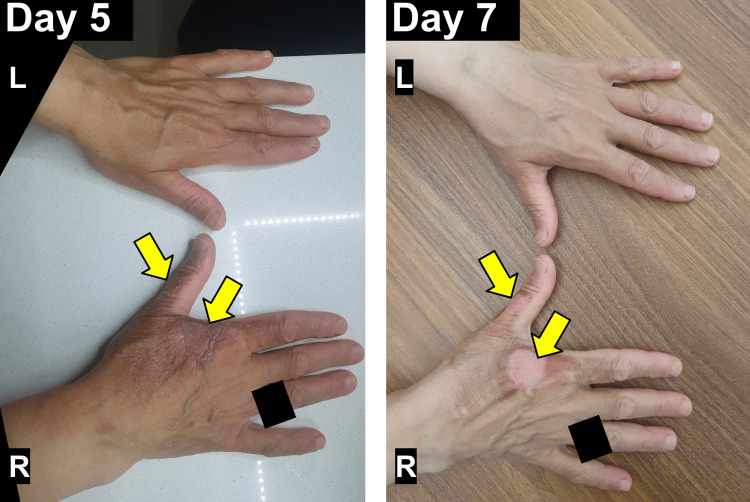
Superficial Partial-Thickness Hand Burn (Days Five and Seven Post-Incident). The superficial partial-thickness hand burn injury on days five and seven post-incident can be seen on the dorsal aspect of the right hand (R=right hand, L=left hand). The blisters at the base of the first and second fingers ruptured on day five by themselves (yellow arrows). Debridement of the popped blisters was performed on day seven (yellow arrows). On day seven post-incident, the previously blistered areas can be seen beginning to re-epithelialize, forming a thin, transparent layer of new skin. Identifying features of the patient, including clothing and rings, have been blacked out upon their request.

She was extremely concerned as the swelling had not been present before she went to sleep, which is when she decided to contact a family friend who happened to be a medical doctor. She was advised to gently apply an antibacterial cream to the burn injury, taking care not to rupture any of the blisters, and to wrap the affected area with a non-adherent sterile dressing so as to protect the wound, keep the area moist, and promote healing, making sure to change the dressing at least once a day. The patient already had fusidic acid cream on the hand, so she applied it to her burn injury and wrapped the area with sterile non-adherent dressings daily as instructed. She also took a daily over-the-counter, non-steroidal, anti-inflammatory drug for pain relief. The blisters at the base of the first and second fingers ruptured on day five by themselves, and debridement of the burst blisters was performed on day seven (Figure 2).

**Figure 3:**
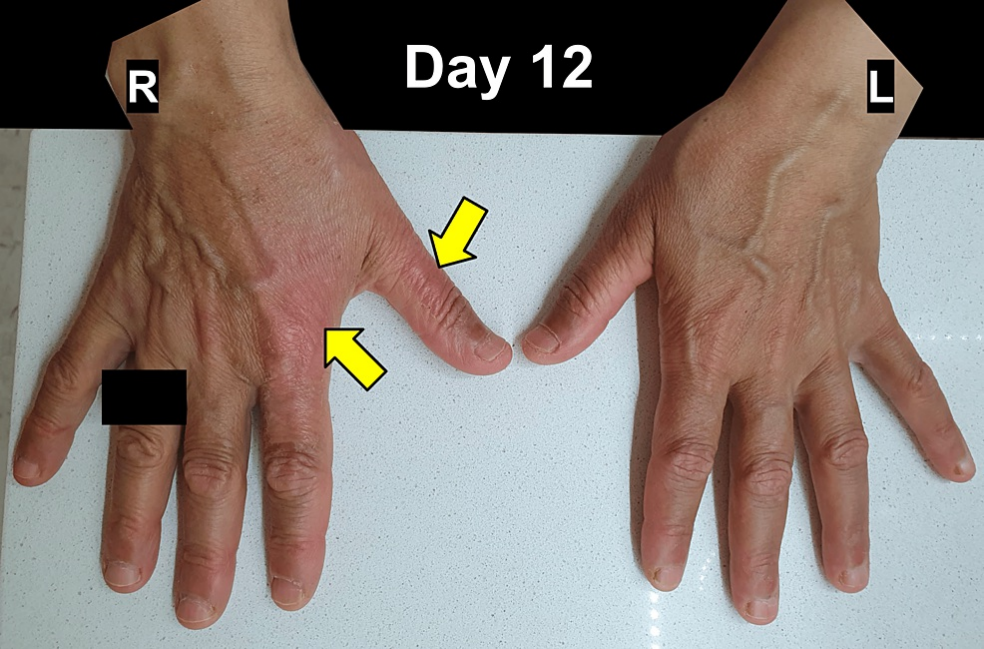
Superficial Partial-Thickness Hand Burn (Day 12 Post-Incident). This image documents the advanced healing stage of a superficial partial-thickness burn on day 12 post-incident of the dorsal aspect of the right hand (R=right hand, L=left hand). The burn site shows signs of re-epithelialization, with the formation of pink, healthy granulation tissue (yellow arrows). The previously observed blisters have resolved completely, and the surrounding skin appears normal. Identifying features of the patient, including clothing and rings, have been blacked out upon their request.

By day 12 post-incident, the previously observed blisters had completely resolved, and signs of re-epithelialization could be seen as the site of the burn continued to heal (Figure 3).

This was a classic case of a superficial partial-thickness thermal burn of the hand as evidenced by the presence of swelling, blisters, and intense pain. It is frustrating that the patient had to go through such an experience especially because the burn could have been easily prevented by wearing oven mitts. This case report aims to emphasize the significance of understanding the potential risks associated with everyday activities involving heated objects, surfaces, or vapors, as well as the importance of implementing preventive measures to minimize the occurrence of burn injuries. By presenting a real-life scenario, we underscore the need for public awareness campaigns, safety guidelines, and educational initiatives aimed at reducing burn injuries that could otherwise be easily prevented. 

## Discussion

Burn injuries are frequently encountered and can originate from a range of sources, such as friction, extreme temperatures, radiation, chemicals, or electricity [1]. Nevertheless, thermal burns caused by contact with hot liquids, solids, steam, or fire remain the prevailing cause of such injuries [1]. While all burn injuries result in tissue damage caused by the transfer of energy, various causes can elicit distinct pathophysiological as well as physiological responses [1]. A burn caused by a flame, for instance, typically results in an instantaneous and deep injury, whereas a burn caused by steam or hot liquids (also referred to as scald injuries) often manifests initially as superficial due to the rapid dispersion and dissipation of the heat source and energy [1]. Interestingly, cold exposure can also result in thermal injury [1]. Direct damage to cells caused by the crystallization of water within tissues, as well as indirect injury stemming from ischemia and subsequent reperfusion, can result in frostbite, leading to deep tissue damage and necrosis of the skin [1]. 

While certain hand burns may be mild in nature, others can be severe and necessitate treatment at a specialized burn center [2]. Even with access to optimal treatment and rehabilitation, a number of patients still encounter debilitating challenges, such as contractures, hypertrophic scars, and neuropathy, that significantly impact their well-being [4]. While hand burns may not directly result in the patient's fatality, they can lead to significant biological, social, and psychological ramifications that diminish the overall quality of life [11]. Nearly 18 million individuals worldwide are believed to experience substantial limitations in their daily lives as a result of hand burns, leading to significant impairments [12]. In addition to the essential aspect of restoring hand functionality, it is crucial to recognize the significance of hand aesthetics, as hands are consistently exposed and actively involved in numerous social interactions [11]. A core principle in modern burn care is that a significant portion of burn injuries can be prevented through effective preventive measures [5]. Establishing a comprehensive nationwide burn prevention education program is crucial, encompassing key areas, such as promoting water temperature control in households, advocating for the use of anti-burn equipment, and encouraging the replacement of outdated heating equipment [3]. 

Besides identifying the cause of a burn injury, it is crucial to classify the injury based on its severity [13]. Burns are categorized based on both the depth of the burn and the extent of the total body surface area affected [6]. A burn can be placed in one of five classifications based on progressive depth: superficial, superficial partial thickness, deep partial thickness, full thickness, and deep full thickness [7]. Burns that only affect the epidermis are classified as superficial (first-degree/grade 1) burns [7]. These burns result in redness as well as desquamation of the skin and present with mild short-lived pain [7]. Superficial partial-thickness (second-degree/grade 2A) burns affect the epidermis and the upper third papillary layer of the dermis [7]. These burns typically result in edema and blister formation and present with moderate to severe pain [7]. These burns may leave minimal scars, but generally do not necessitate surgical intervention [7]. Deep partial-thickness (second-degree/grade 2B) burns on the other hand affect the entirety of both the epidermis and the dermis and are notably less painful due to partial destruction of pain receptors [7]. Both blisters and eschar may be present [7]. Full-thickness (third-degree/grade 3) burns extend through the entire dermis to the subcutaneous tissue, are typically not painful due to nerve damage, require topical antimicrobial protection against infection, and, unless they are very small, often necessitate surgical management and skin grafts [7]. These burns may appear brown to black and eschar formation may be observed [7]. Lastly, a deep full-thickness (fourth-degree/grade 4) burn involves damage to deeper tissues such as muscles or bones, often appears blackened, and frequently leads to the loss of the affected body part [7]. These burns are the most severe and often present with substantial impairment, both in terms of functionality and appearance [7]. The percentage of total body surface area is also utilized to classify burns [6]. Burns that involve less than 20% of the total body surface area are categorized as local burns, while those that affect more than 20-30% of the body are classified as severe burn injuries (SBI) [14]. SBI can lead to systemic disturbances that necessitate intensive management [14]. 

The treatment approach for a burn injury depends on its specific cause [1]. For instance, immediate surgical intervention, debridement, and grafting may be required for deep thermal burns; however, applying the same approach to frostbite would be a mistake [1]. For the purposes of this case report, we will focus on the treatment of second-degree, superficial, partial-thickness burns. The management of a superficial partial-thickness burn on the hand begins with wound care [8]. The burned hand is gently cleaned with a mild, sterile saline solution or clean water to remove debris and contaminants [8]. Care should be taken not to scrub the wound or cause further damage [8]. Blisters that have formed are typically left intact to protect the underlying skin and promote healing [8]. Popping or draining blisters should be avoided by the patient [8]. Topical antimicrobial agents, such as silver sulfadiazine, bacitracin, mupirocin, and fusidic acid, among others, may be applied to the burn wound, alone or in combination with each other [9,10]. This helps prevent infection and provides a moist wound-healing environment [15]. There is currently no widely agreed-upon consensus regarding the most effective agent or dressing for the management of burn wounds, specifically in terms of preventing or controlling infection and promoting wound healing [16]. Topical treatments, such as aloe vera and honey, as well as fish skin-based wound dressings, are some of the treatment modalities currently being explored for potential use in the management of superficial partial-thickness burns [17]. Pain medication, such as over-the-counter analgesics (e.g. acetaminophen or non-steroidal anti-inflammatory drugs), may be recommended to manage pain and discomfort [18]. Topical anesthetics, such as lidocaine, may be used to alleviate local pain and provide temporary relief [18]. Non-stick or non-adherent dressings are commonly used to cover the burn wound [10]. These dressings help protect the wound, minimize pain during dressing changes, and promote healing [10]. Regular dressing changes are essential to create a bacteria-free environment, support wound healing, and facilitate painless removal of dressings [10]. The frequency of dressing changes may vary based on the type of dressing employed, ranging from as often as every 12 hours to weekly [10]. Regular follow-up visits with a healthcare professional are scheduled to monitor the progress of wound healing, assess for complications, and adjust the treatment plan as needed [19]. Rehabilitation, including occupational therapy or hand therapy, may be recommended to promote functional recovery, prevent contractures, and improve range of motion [19].

## Conclusions

This case report highlights the occurrence of a superficial partial-thickness burn to the dorsal aspect of the hand resulting from sudden unexpected exposure to intense hot air emitted by a convection oven. We emphasize the importance of understanding the risks of thermal burns from everyday activities involving heated objects and how the hand burn in this case could have easily been prevented by the use of oven mitts. We discuss the importance of prompt medical attention and proper wound care in helping mitigate the severity of burn injuries through the use of antimicrobial agents and non-adherent dressings. While the findings may not be groundbreaking, this report contributes to the medical literature by providing insights into the management of accidental burns caused by convection ovens. Moreover, it raises awareness among healthcare professionals and the public about potential risks associated with common household appliances. Documenting such cases can help establish a comprehensive body of evidence and facilitate future research in burn injury patterns. Advancing our knowledge in this area can lead to a reduction in avoidable burn injuries and improve the quality of care for those affected.
